# Collagenolytic Matrix Metalloproteinases in Chronic Obstructive Lung Disease and Cancer

**DOI:** 10.3390/cancers7010329

**Published:** 2015-02-05

**Authors:** Denzel Woode, Takayuki Shiomi, Jeanine D’Armiento

**Affiliations:** Department of Anesthesiology, Columbia University, College of Physicians and Surgeons, New York, NY 10033, USA; E-Mails: drw2118@cumc.columbia.edu (D.W.); ts2425@cumc.columbia.edu (T.S.)

**Keywords:** collagenases, COPD, lung cancer, cigarette smoke

## Abstract

Chronic obstructive pulmonary disease (COPD) and lung cancer result in significant morbidity and mortality worldwide. In addition to the role of environmental smoke exposure in the development of both diseases, recent epidemiological studies suggests a connection between the development of COPD and lung cancer. Furthermore, individuals with concomitant COPD and cancer have a poor prognosis when compared with individuals with lung cancer alone. The modulation of molecular pathways activated during emphysema likely lead to an increased susceptibility to lung tumor growth and metastasis. This review summarizes what is known in the literature examining the molecular pathways affecting matrix metalloproteinases (MMPs) in this process as well as external factors such as smoke exposure that have an impact on tumor growth and metastasis. Increased expression of MMPs provides a unifying link between lung cancer and COPD.

## 1. Introduction

Chronic obstructive pulmonary disease (COPD) and lung cancer are both significant causes of morbidity and mortality in the United States and worldwide. It is estimated that approximately 400,000 deaths occur each year due to COPD [1], and there is a 15.0% 5-year survival rate for late stage lung cancer patients, a point of time when 85% of lung cancer is diagnosed [2]. Recent studies have shown that both COPD and lung cancer share a similar genetic pre-disposition [3]. It is well understood that patients diagnosed with COPD have a higher risk of lung cancer [4], and the presence of computed-tomography diagnosed emphysema is linked to a lower survival rate among patients with early onset lung cancer [5]. The incidence of COPD increases the risk of lung cancer up to 4.5 fold, with the onset of lung cancer associated with a greater severity of COPD [3,6]. This poor prognosis for lung cancer is associated with reduced lung function seen in patients with COPD. Emphysema is the result of lung destruction and is a component of the complex phenotype of COPD. The diagnosis of emphysema is characterized pathologically [5,7] with the forced expiratory volume in 1 second (FEV_1_) serving as a proxy for a patient’s lung damage. Epidemiological evidence has demonstrated that a low FEV_1_ is associated with an increased risk of lung cancer in middle aged white males [8]. These interesting associations suggest that COPD and lung cancer potentially have overlapping pathological processes.

Cigarette smoke is the major common etiologic agent that activates mechanisms leading to the development of COPD and lung cancer [9]. Cigarette smoke constituents induce inflammation and oxidative stress in lung tissue [10,11], which alters the transcription and activation of proteases generating an imbalance of proteolytic enzymes and their inhibitors [12] within the parenchyma of the lung. The imbalance between proteinases and their inhibitors, defined as the proteinase-anti-proteinase theory, in addition to genetic modulation, causes damage to the lung tissue [11] and could predispose to lung cancer progression.

Matrix metalloproteinases (MMPs) are the major proteinases responsible for the digestion and degradation of structural proteins of the lung [13]. Fibrillar collagen is a critical extracellular matrix (ECM) molecule, produced by various stromal cells, that provide the material necessary for the cellular organization of the lung, and comprises 60% of lung protein [14]. Collagenases (MMP-1, MMP-8 and MMP-13) can digest major fibrillar collagens at the triple-helical domain [15]. The digestion of the collagen fibril is an essential step in the remodeling of tissue architecture *in vivo*. MMP-8 is the major collagenase produced in neutrophils and plays important roles in the acute phase of the inflammatory process, while MMP-1 and MMP-13 are thought to be vital in physiological tissue remodeling and chronic tissue remodeling under various pathological conditions [15].

The expression of MMPs is tightly regulated at the transcriptional level by extracellular stimuli, hormones, growth factors, cytokines, cell-cell and cell-ECM interactions and transformation [16]. Our laboratory has demonstrated that lung parenchymal cells in patients with emphysema express MMP-1, as opposed to smokers without the disease [17], and through *in vitro* and *in vivo* studies, we demonstrated that cigarette smoke can directly induce MMP production in epithelial cells in a MAP Kinase dependent fashion [18]. Subsequent studies identified a novel cigarette smoke responsive (CSR) element within the promoter region of MMP-1 [19]. We further delineated the upstream signaling pathway regulating MMP-1 induction by cigarette smoke and identified Toll-Like Receptor 4 (TLR4) as an important regulator of the induction of MMP-1 [20]. TLR4s are classically involved in innate and adaptive immunity through their recognition of pathogen associated molecular patterns [21]. Recently, studies have shown that TLR4s are activated by cigarette smoke in mice and rabbits, and have a role in the regulation MMP-1 production, oxidative stress, and autophagy in lung cells, [20,22].

**Figure 1:**
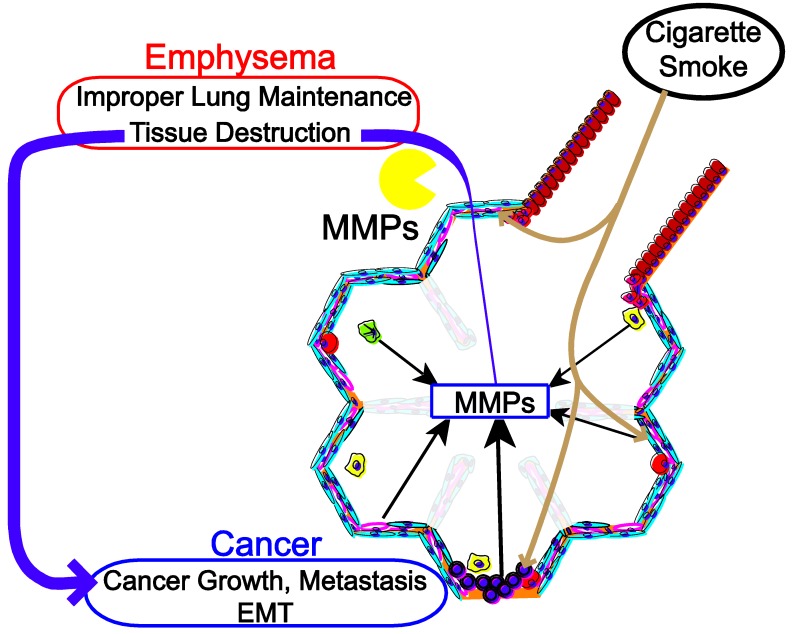
High MMPs microenvironment to promote and sustain emphysema and cancer progression.

It is our hypothesis that the modulation of molecular pathways that are activated during emphysema and lead to protease production predispose the lung to tumor growth and metastasis. The present review presents *in vitro* and *in vivo* studies exploring the modulation of biological pathways that influence activation and up regulation of MMPs, promote tumor growth, metastasis, and increase the likelihood of developing COPD and emphysema in both mice and humans (Figure 1).

## 2. Lung Diseases and Collagenases

### 2.1. COPD and Collagenases

MMP-1, MMP-8, and MMP-13, are members of the MMP sub family of collagenases, and each has been implicated in the development of COPD in response to cigarette smoke both *in vitro* and *in vivo*. As described above, our laboratory has demonstrated a major role for MMP-1 in the development of emphysema [17,19,23,24,25]. Genetic studies have also supported the increase in MMP-1 observed, as *in vitro* studies have shown an increase in MMP-1 promoter activity in the presence of 5% cigarette smoke extract (CSE) [19,25].

Early animal studies directly demonstrated that increased local expression of MMP-1 promotes emphysema and lung degradation in transgenic mice expressing the human MMP-1 gene [23] within lung parenchymal cells. Further, when MMP-1 was expressed in the epidermis of transgenic mice, hyperproliferation of cells occurred in response, resulting in hyperplasia and acanthosis of the skin [26]. Skin tissue and lung tissue appear to have different responses to the overexpression of collagenase, as the skin has the ability to regenerate cells which would have resulted in the hyperplastic lesions observed in the study [26], whereas the repair of lung cells is more restricted, and resulted in emphysema as a result of alveolar destruction [27]. Subsequently, MMP-1 protein, mRNA expression, and proteolytic activity were detected in the lung parenchyma of emphysema patients, and not in normal healthy patients [17], supporting the results of the animal studies and establishing MMP-1 as a fundamental degradative enzyme in the destructive disease of emphysema.

MMP-13, another member of the collagenase family, was recently discovered to be increased in alveolar macrophages and type II pneumocytes of COPD patients [28], and its expression was also found to correlate with shedding and aggregation in the bone marrow in non-small cell lung cancer patients, the effects of which are coincide with poorer survival rates [29]. MMP-13 mRNA is increased due to short term smoke exposure in the muscularized small intrapulmonary arteries in mice, through induction of the tumor necrosis factor alpha (TNF α) pathway [30]. Cigarette smoke increases TNFα *in vitro*, through the activity of necrosis-factor kappa B (NF-κB) transcription factor [31], which is released by macrophages that are recruited to the lungs in response to cigarette smoke, and is therefore a major regulator of the protease imbalance after cigarette smoke exposure [12].

Proteolytic enzymes synergistically degrade the ECM and expression of one protease can then lead to the induction and/or activation of other proteases. MMP-13 activates proMMP-9 *in vitro*, which can result in the continued degradation of the ECM *in vivo* [32]. In addition, degraded collagen molecules denature gelatin, which is cleaved into smaller peptides by gelatinases, such as MMP-9 [33,34]. It is highly likely that the overall mechanism of destruction in COPD and lung cancer involves multiple proteases acting through intricate interactions with each other, their specific inhibitors, and their matrix substrates [14,35,36,37,38,39]. Our lab established a role for MMP-9 expression in the progression of lung degradation and the development of emphysema by expressing human MMP-9 in the macrophages of transgenic mice [40].

MMP-8 has also been implicated in the development of progressive lung diseases. Expression of MMP-8 and MMP-9 are up regulated in the induced septum of COPD patients [41], and MMP-8 localizes with neutrophils within the alveolar septa and alveolar spaces of the lung tissue in COPD patients [42]. The up regulation and localization of MMP-8 in neutrophils was associated with an increased presence of MMP-1 in the interstitial and alveolar macrophages [42]. The increased presence of inflammatory cells causes oxidative stress in the lung, which in addition to the increase in proteolytic activity from collagenases and other metalloproteinases, likely results in the development of COPD, and predisposes the lung to other insults.

### 2.2. Cancer and Collagenases

These same enzymes that carry importance in emphysema, have a vital role in tumor invasion and metastasis [14,35,36,37,38,39,43]. MMPs have been studied and implicated in the development of increasingly aggressive cancer cells, due to the proteolytic activity of MMPs and their degradation of the structure of tissue barriers [14]. The degradation of the ECM is a critical step in tumor development, as it leaves damaged surrounding tissue that can be used as a scaffold for migration for metastatic tumor cells [44,45]. Studies have shown that high levels of MMP-9 are associated with poorly differentiated non-small cell lung cancer [36]. Another study provided evidence that low levels of MMP-9, both *in vivo* and *in vitro*, allowed for increased tumor angiogenesis; the vascularization of tumor cells is essential for tumor growth [37]. This same study also revealed that high levels of MMP-9 *in vivo* exhibit an anti-tumorigenic effect by producing angiostatin, an inhibitor of angiogenesis [37]. Tumor associated macrophages (TAM) also release MMP-9, and the increase in MMP-9 can result in the release of matrix-sequestered vascular endothelial growth factor-A (VEGF-A) [39,46]. Studies have shown that VEGF receptor (VEGFR) positive distal organs are more likely to become metastatic sites for cancer cells [39,47].

More specifically, recent data supports the notion that an elevated presence of collagenases is associated with an increase in the severity of cancer. A specific MMP-1 allele, the 2G allele, is associated with a higher risk of lung cancer in individuals who have never smoked as well as in patients with the adenocarcinoma subtype of non-small lung cell cancer [38]. It is possible that the different derivatives of the MMP1 allele result in varying levels of transcription and expression of MMPs in the cellular environment, resulting in a greater susceptibility to cancer in patients with the 2G allele. Our lab has shown that increased MMP-1 synthesis can predispose the cellular environment to tumor development and tumor aggressiveness, in addition to its central role in emphysema [26]. Patients with COPD are likely more predisposed to lung cancer, due to the increased presence of MMPs and MMP promoter activity caused by the presence of cigarette smoke elements [19,38] The consistent interaction of MMPs with the cell surface allows proteinases to specifically target points where tumor cells are in contact with the ECM, which can improve the survival and migration potential of tumor cells [14]. MMP-1 interacts with the tumor cell surface through an extracellular matrix metalloproteinase inducer (EMMPRIN), suggesting that the binding and activation of MMP-1 by EMMPRIN may be a significant step in the development of aggressive tumor cells [14,44,48]. It has also been shown that the up-regulation of membrane-type 1 matrix metalloproteinase (MT1-MMP) cleaves and activates EMMPRIN, further supporting the notion that the activation of one specific MMP can result in the release of others [45].

The degradation of the ECM surrounding tumor cells is an intricate, complex process that likely involves multiple proteinases during tumor development. In addition to MMP-1, studies have identified MMP-13 as a collagenase that is associated with a high risk of lung cancer [29,33,49,50,51]. MMP-13 is critical for angiogenesis in wound tissue in mice [51], suggesting that an increased presence of MMP-13 leads to greater vascularization. In addition to this data, an increase in the presence of MMP-13 is associated with malignant tumors, and the transformation of benign tumors to malignant [50]. A possible explanation is that tumor cells stimulate the production of MMP-13 in adjacent cells, as high levels of MMP-13 mRNA were observed in stromal cells adjacent to MCF-7 breast cancer cells *in vitro* [52]. Induction of MMP13 production by breast cancer cells has also been shown to facilitate metastasis [45,53].

MMPs also degrade the ECM in a fashion that allows tumor cells to be released from binding factors in their environment, and increase the motility of the cells [14]. MMPs appear to predispose the sites to cancer, in part by providing areas for endothelial cells to migrate and form new fixtures for blood vessels, and by also releasing a variety of ECM sequestered signaling factors from the cell surface [14], which may stimulate growth pathways of the cell resulting increased cell proliferation [26]. The expression of MMP-13 has been associated with the presence of tumor cells in the bone marrow of non-small lung cancer patients, and a reduced prognosis compared to MMP-13 negative patients [29]. Although bone marrow micro-involvement is not fully associated with the likelihood of tumor metastasis [54], it is a strong indicator of tumor progression and of a worsening prognosis [29]. In addition, data correlates the presence of collagenases and their transcripts with a poorer prognosis of cancer [3,5,14,50,55].

MT1-MMP has been shown to induce the epithelial-mesenchymal transition (EMT) in several carcinomas [45]. EMT is an indicator of tumor progression and the development of metastasis, and is a process by which cells transition from an epithelial to a mesenchymal phenotype [3], and is essential for tumors to develop motility and invasive characteristics [56]. EMT is significant in both lung cancer and COPD progression due to the significance of cell migration in the progression of both diseases [3,56,57]. MMPs can degrade E-cadherin and release cytokines and transcription factors that reduce the E-cadherin expression, and consequently induce EMT [56]. A recent study shows a correlation between MMP-1 expression and circulating breast cancer tumor cells and a relationship between MMP-1 expression and high tumor cell proliferation [55]. Further studies on MMP-1 involvement in EMT may reveal and important mechanistic step in the development of tumor cells metastatic potential.

## 3. Cigarette Smoke in COPD, Lung Cancer through the Regulation of Collagenase 

Epidemiological studies suggest a connection between the development of COPD and lung cancer in addition to the role of environmental smoke exposure in the development of both diseases [3]. There is a known positive correlation with a previous diagnosis of COPD, emphysema, and the development of lung cancer [58], with further studies showing that the presence of emphysema is associated with a lower likelihood of survival in lung cancer patients [5]. Cigarette smoke leads to alters gene expression within the epithelial cells of the lung leading to lung damage and aberrant lung function [10,59,60]. Joos and colleagues showed that the expression of specific MMP-1 alleles was associated with faster decline in lung function in smokers developing emphysema [61]. Furthermore, it is known that reduced pulmonary function then increases the risk of both COPD and lung cancer [3]. Collagenases can alter lung function in both COPD and lung cancer by destroying the alveolar wall structure and releasing cytokine and growth factors that could stimulate tumor growth [56]. MMP-1 alleles have also been associated with the presence and increasing severity of different cancer lines [61,62,63,64].

It is known that there is an increase in the expression of interstitial collagenase in patients with emphysema [65]. This can be attributed to the physical damage of the lung caused by the cigarette smoke, which not only increases proteases within the lung parenchyma, but also results in the recruitment of immune cells that produce MMPs [12,31].

It is likely that the augmentation and prolonged presence of MMPs could result in the modification of mechanisms in the lung that results in the development of tumors and COPD. The continued degradation of the ECM releases cytokines and chemokines from the ECM. The epithelial cytokine, CXCL14, is up regulated *in vitro* by the presence of CSE, and *in vivo* in patients with smoke induced COPD, and further elevated in patients with non-small cell lung cancer [66]. The most elevated levels of CXCL14 were observed in areas in which lung cells had undergone EMT [66]. EMT is both observed in COPD and lung cancer, and the increased presence of MMPs may be a result of the degradation of E-Cadherin; the soluble form of E-Cadherin that is then released further stimulates and increases the production and release of MMPs [67].

## 4. Limitations of Collagenase Research

One current restriction with research on MMPs is the lack of animal models that naturally produce murine MMP-1 with homologous functionality to human MMP-1. MMP13 is the main natural collagenase studied in rodent models. Although mouse orthologues of MMP-8 and MMP-13 have been identified and studied [68,69], mice lack expression of a MMP-1 gene that confers a protein with the same specificity as the human MMP-1 [70]. Murine proteinases Mcol-A (MMP-1a) and Mcol-B (MMP-1b) have been described as orthologues of human MMP-1 [70]. Mcol-A was found to be the most likely to have homologous functions to human MMP-1 [70]. However, our lab has recently shown that these novel proteinases did not respond to cigarette-smoke extract (CSE) *in vitro* [71], unlike human MMP-1 which is significantly up regulated by CSE, thus reducing the possibility of using the expression of MMP-1a or MMP-1b in cigarette smoke models. The varying responses to CSE may be due to the differences in the distal 1 kb promoter region observed by our lab (Carver *et al.* under revision) [19]. Further research needs to be completed to find a natural homologue to human MMP-1 in murine models of cigarette smoke.

Research on the collagenases has focused on the digestion of ECM substrates, such as collagen. Collagenases also have important non-ECM substrates. MMP-1 and MMP-13 non-ECM substrates include α1-antichymotrypsins and CTGF; MMP1 specific non-ECM substrates include α2-M, α1-P1, IGFBP-2,3,5, and proIL-1β; MMP-13 specific substrates include pro-TGF-β, and MMP-8 specific non-ECM substrates include α1-PI [13]. Potentially, the collagenases act through non-ECM mechanisms that have an effect on the development of COPD and lung cancer. In addition, MMP-1 directly activates protease-activated receptors (PAR) on the breast cancer cell surface, which results in pro-invasive signals [72]. Tumor-derived MMP-1 has also been shown to activate PAR, resulting in acute endothelial cell activation, and pro inflammatory signals [73]. The physiological changes that occur can increase the metastatic potential of tumor cells, thus providing an alternative mechanism where collagenases influence the progression of cancer cells.

Due to the pro-inflammatory and pro-carcinogenic signals MMPs modulate, research has focused on the development of synthetic MMP inhibitors (MMPi) as therapeutic drugs that could prevent ECM degradation, thus inhibit the development of COPD and cancer. However, clinical trials using MMPi for cancer treatment have generally not provided positive results, some even resulting in a poorer survival rate [14]. One possible issue with current synthetic inhibitors is the fact that they do not target a specific, individual MMP, but cover a broad number of family members. Research has now shown that MMPs, as regulators of cytokine and growth factor availability, have a wide and complex set of roles that can also be protective in certain cancers, instead of protumorigenic [74]. Thus, non-specific inhibition of MMPs can lead to a poorer prognosis. Development of an MMPi that is selective for a specific collagenase is still a potential approach to treat cancer patients [14]. Advances in technology allow for the development of new synthetic MMPi’s that specifically target single MMP family members are most relevant in the respective line of cancer research [74].

## 5. Future Directions

The expression of collagenases is vital in the pathogenesis of both lung cancer and COPD, and the development of better animal models is critical for future advances in research. It is important to create a more reliable transgenic mouse model to be utilized for cigarette smoke studies that will effectively document the induction of collagenase within the lung parenchyma by cigarette smoke in order to better understand the downstream effects of this induction.

Clinically, collagenases can still serve as potential therapeutic targets for progressive chronic lung diseases. MMP-1 and MMP-13 should continue to be looked upon as targets in cancer and COPD therapy. Highly selective inhibitors of MMP-1 and MMP-13 could down regulate the expression of these collagenases, decreasing the activation and release of other signaling factors and proteinases, thus resulting in a more successful therapy. Naturally derived MMPi could also be isolated and utilized to target MMP-1 and MMP-13, which may reduce the likelihood of unwanted interactions and improve results. Most importantly, factors upstream of MMP-1 and MMP-13 regulation can serve as potential targets of inhibition and result in a better prognosis without interrupting vital pathways the cell requires for healthy ECM interaction. One such target would be the TLR-4 signaling pathway. We have shown TLR-4 regulates the expression of MMP-1 upon cigarette smoke; furthermore, the lungs from smokers exhibit elevated levels of TLR-4 and MMP-1 [20]. Additional regulators of the TLR-4 smoke signaling pathway may unveil new therapeutic targets that can be inhibited to down regulate the expression of MMPs in the presence of cigarette smoke, which would likely be beneficial in the treatment of both COPD and lung cancer.
